# *Cousinia waldheimiana* (Asteraceae) a new record from Uzbekistan (Central Asia)

**DOI:** 10.3897/phytokeys.77.11897

**Published:** 2017-03-17

**Authors:** Mansur X. Usmonov

**Affiliations:** 1 Central Herbarium, Institute of the Gene Pool of Plants and Animals of Academy of Sciences of the Republic of Uzbekistan, P.O. Box st. Durmon yuli 32, 100125, Tashkent, Uzbekistan

**Keywords:** Asteraceae, *Cousinia* sect. *Jurineopsis*, Uzbekistan, Kyrgyzstan, Alay

## Abstract

During the study of plant specimens in the Central herbarium Academy of Sciences of Uzbekistan Institute of the gene pool of plants and animals (TASH) one new record for the flora of Uzbekistan (*Cousinia waldheimiana*) was identified. This species is the fourth member of *Cousinia* sect. *Jurineopsis* collected in the Uzbek part of the Northern Alay range.

## Introduction

The genus *Cousinia* Cass. with ca. 600-700 species (Attar et al. 2001; Assadi 2010; Attar and Maroofi 2010; Mehregan et. al. 2010) is the second largest genus of Asteraceae, after *Senecio* L. (Memariani and Joharchi 2011). Cousinia (tribe *Cardueae*) is subdivided into three subgenera (Tscherneva 1962, 1974) and ca. 70 sections (Kadereit and Jeffrey 2007, Mehregan and Kadereit 2009). The genus is unique for its high level of diversity and penchant for narrow-range endemism. (Tscherneva 1974, Rechinger 1986). Eight major centers of species diversity have been defined for the genus in South West Asia and Central Asia (Mehregan 2008). The most important center of species diversity is situated in Pamir-Alay and Tien Shan ranges in the Central Asia with ca. 230 species, of which 150 are endemics (Tscherneva 1974). The Turkman-Iranian mountainous province in North East Iran and South Turkmenistan can be considered as the second important center of diversity for *Cousinia*, with approximately 100 species, of which 70 species are considered endemic to the area (Rechinger 1972, 1979).

As part of the project “Botanical and geographical regionalization of Uzbekistan and creation of a database of plant diversity. Part II. Turan province” herbarium specimens stored in TASH of the genus *Cousinia* were processed. The process of working with more than 3000 specimens showed that some specimens had not been previously recorded for some Central Asian countries. This paper presents the results of processing the specimens relating to Cousinia sect. Jurineopsis (Juz.) Tscherneva.

Cousinia sect. Jurineopsis is an endemic section of 11 species found in the Central Asia Mountains (Tscherneva 1993). Earlier Tscherneva (1962) recognized three species - *Cousinia dubia* Popov, *Cousinia krauseana* Regel et Schmalh., *Cousinia submutica* Franch. for this region in the flora of Uzbekistan. During the processing of the specimens in this section at TASH a further species *Cousinia waldheimiana*, hitherto known only from outside of the Uzbek borders in North East Kyrgyzstan (Central and Western Tien Shan), was identified. This is a new record for the flora of Uzbekistan.

## Materials and methods

The identification was based on consultation of published accounts of *Cousinia* (Tscherneva 1974; Zare et al. 2012; Mehregan and Kadereit 2008; Sennikov 2010, 2011; Mabberley 1990; Heffner 2000; Susanna et al. 2003; Djavadi 2012; Sheidai et al. 2006) and herbarium specimens stored in the TASH.

## Results

### New record

#### Cousinia Waldheimiana

Taxon classificationPlantaeAsteralesAsteraceae

Bornm. 1916, Beih. Bot. Centrl. XXXIV. II. 140. (1916).

urn:lsid:ipni.org:names:199419-1

Fig. 1

##### Description.

Biennial plant. Stem erect, 30–80 cm tall, subglabrous , basally covered in brownish thinly-arachnoid hairs; branches elongated, mid-corymbosa, patulous, monocephalous. Leaves coriaceous, glabrous above, green, tomentose below; basal leaves pinnatisected, cauline sessile, semiamplexicaul, narrowly lanceolate or lanceolate, drawn to the apex, almost smooth-edged, just at the base of usual with 2-4 pairs of teeth; branches of the leaves strongly diminished. Capitula ovate-conical, slightly arachnoid, 2.5–3 cm in diameter, (without acidotus) monocephalous. Phyllaries are numerous in number 90–100, except internal, with a slightly arcuately reflect acidotus; internal protruding up, lanceolate; scarious on top, acerous in a very thin, short the barb, usually brownish; setula receptacle above advanced, asperous. Corolla pink. Achenes ca. 3.5–4.0 mm long, obovate, glabrous and smooth.

##### Phenology.

Flowering from June to July, fruiting from July to August.

##### Habitat.

On the rubbly-fine earth slopes, shale rocks in the middle belt of mountains.

##### Distribution.

Previosuly considered endemic to the Western Tien Shan Mountains to distribution in Chatkal, Fergana, Uzunakhmat and Atoynak ranges. This range is now extended to the Alay Range, Uzbekistan (Fig. 2).

##### Specimens seen.

Uzbekistan. Alay Range (North side): Fergana Valley, above the Shakhimardan resort on the slopes of the river Jordan. 02.08.1954, *Korotkov 4569* (TASH); Alay Range. River basin Shakhimardan, neighborhood of village Jordan. Stony NE slope of the upper terrace of the right bank of the river. Ak-su. Tree and shrub belt. 16.07.1961, *Pyataeva 118* (TASH); S slope of the Alay range. River basin. Shakhimardan, neighborhood of village Jordan. 1600 m. NE slope of the right bank of the river. Ak-suv. 16.07.1961, *Abdullayev 66* (TASH).

##### Other specimens examined.

Kyrgyzstan. Jalal-Abad region. Kyrg. SSR. Upper river of the Hodge-ata. On the pass and the lake Sary-Chelek. 31.07.1949, *Bondarenko 1197* (TASH); Central Tien Shan. Ketmen-Tyube region. Valley Uzun-Akhmat. Wormwood steppe. 21.07.1927, *Abolin* 455 (TASH); Central Tien Shan. Ketmen-Tyube region. Ayukty river, the hole Almaly. The stony slope. 2007.1927. *Abolin 414* (TASH); The Ferghana Valley. Fergana mountain range near the village Dmitrievka (river Kugart). 29.06.1955, *Korotkov 4894* (TASH).

##### Species recognition.

Very close to the *Cousinia margaritae* Kult., differing mainly by the color of the corolla (from *Cousinia margaritae* corolla whitish or pale yellow in *Cousinia waldheimiana* corolla pink or purple). However, color of the corolla in the herbarium of poorly stored, making difficulties to define plant, why the geographical boundary of these species are currently unclear (Tscherneva 1962).

**Figure 1.**
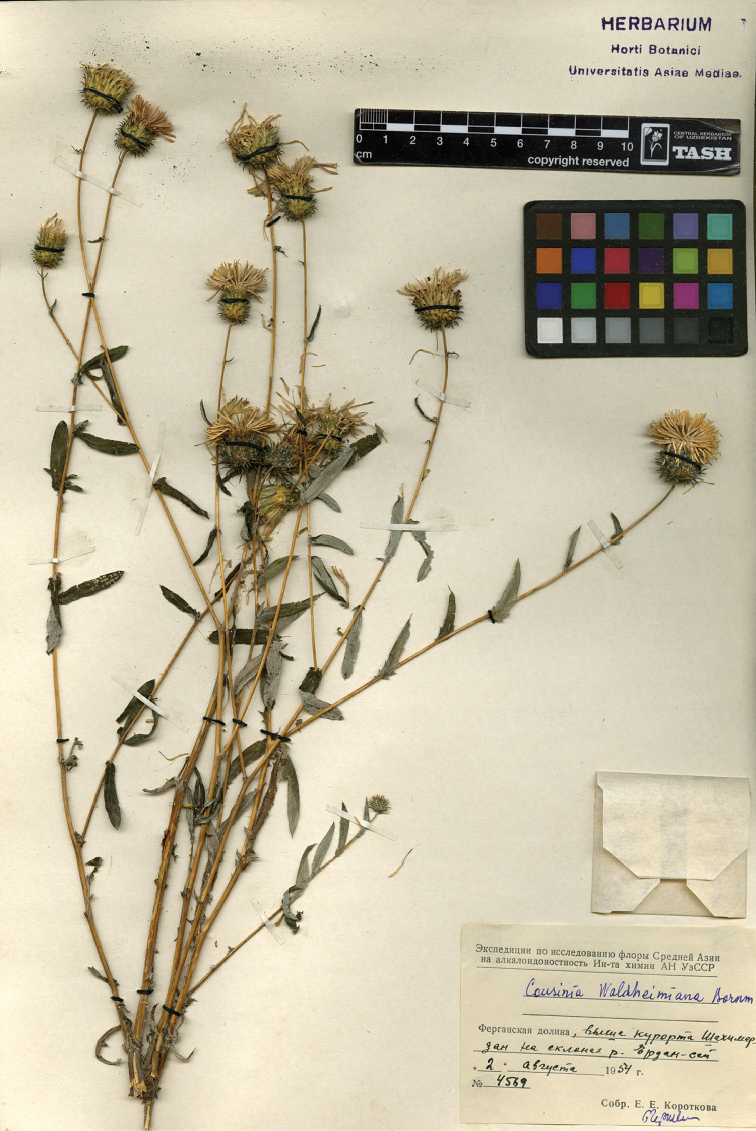
Representative specimen of *Cousinia waldheimiana*.

**Figure 2.**
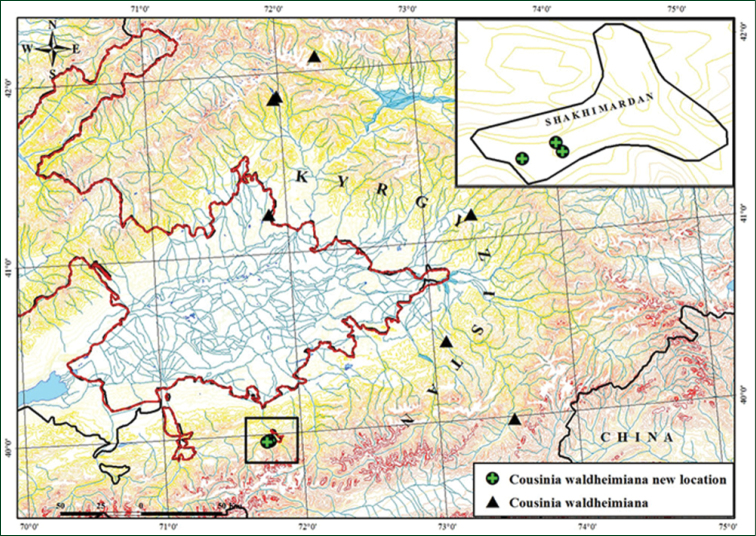
Distribution map of *Cousinia waldheimiana* in Uzbekistan and the neighboring territories (according to the specimens examined).

## Discussion

The species with the areal in Chatkal, Fergana, Uzunakhmat and Atoynak ranges of Western Tien Shan. Its indicated only for the flora of Kyrgyzstan (Tscherneva 1965, 1993). The herbarium specimens from Uzbekistan reported on here were collected from the northern slopes of the Alay range within the Fergana Valley:

## Supplementary Material

XML Treatment for Cousinia Waldheimiana
